# Stress Distribution Analysis on Hyperspectral Corn Leaf Images for Improved Phenotyping Quality

**DOI:** 10.3390/s20133659

**Published:** 2020-06-30

**Authors:** Dongdong Ma, Liangju Wang, Libo Zhang, Zhihang Song, Tanzeel U. Rehman, Jian Jin

**Affiliations:** Department of Agricultural and Biological Engineering, Purdue University, 225 S. University St., West Lafayette, IN 47907, USA; dongdongma812@gmail.com (D.M.); wang3335@purdue.edu (L.W.); zhan2693@purdue.edu (L.Z.); song399@purdue.edu (Z.S.); trehman@purdue.edu (T.U.R.)

**Keywords:** corn leaf, hyperspectral imaging, plant phenotyping, leaf stress distribution, machine learning algorithms

## Abstract

High-throughput imaging technologies have been developing rapidly for agricultural plant phenotyping purposes. With most of the current crop plant image processing algorithms, the plant canopy pixels are segmented from the images, and the averaged spectrum across the whole canopy is calculated in order to predict the plant’s physiological features. However, the nutrients and stress levels vary significantly across the canopy. For example, it is common to have several times of difference among Soil Plant Analysis Development (SPAD) chlorophyll meter readings of chlorophyll content at different positions on the same leaf. The current plant image processing algorithms cannot provide satisfactory plant measurement quality, as the averaged color cannot characterize the different leaf parts. Meanwhile, the nutrients and stress distribution patterns contain unique features which might provide valuable signals for phenotyping. There is great potential to develop a finer level of image processing algorithm which analyzes the nutrients and stress distributions across the leaf for improved quality of phenotyping measurements. In this paper, a new leaf image processing algorithm based on Random Forest and leaf region rescaling was developed in order to analyze the distribution patterns on the corn leaf. The normalized difference vegetation index (NDVI) was used as an example to demonstrate the improvements of the new algorithm in differentiating between different nitrogen stress levels. With the Random Forest method integrated into the algorithm, the distribution patterns along the corn leaf’s mid-rib direction were successfully modeled and utilized for improved phenotyping quality. The algorithm was tested in a field corn plant phenotyping assay with different genotypes and nitrogen treatments. Compared with the traditional image processing algorithms which average the NDVI (for example) throughout the whole leaf, the new algorithm more clearly differentiates the leaves from different nitrogen treatments and genotypes. We expect that, besides NDVI, the new distribution analysis algorithm could improve the quality of other plant feature measurements in similar ways.

## 1. Introduction

High-throughput imaging sensor technologies have been well established for measuring crop growth status in fields or indoors. Plant imaging models have been developed to predict plants’ physiological features, such as the plant’s biomass [1], field yield [2], chlorophyll content [3], nitrogen content [3], relative water content [4,5], and disease symptoms [6,7]. In conventional plant phenotyping analyses, most studies rely on the averaged spectrum across all the segmented plant pixels of the whole plant [2,4,8,9,10,11,12]. Researchers firstly segment the target plant tissue from the image. Then, the averaged spectrum across all the plant pixels is utilized for prediction purposes. However, this conventional phenotyping analysis method is not satisfactory, due to the severe variance of nutrients and stresses across the canopy. For example, it is common to have up to five times of difference among SPAD readings of chlorophyll content at different locations on the same corn leaf [13,14], so that the averaged value cannot characterize the different leaf parts.

At the same time, simply averaging the spectrum information of images and ignoring the distribution features in plants does not fully utilize the collected image data. In fact, the nutrients and stresses have been proven to be nonuniformly distributed in the plant’s body, both vertically [15] and horizontally [10]. For example, the N, P, L, Ca, and Mg of the grain and the residue in the same corn plant were found to be very different [16]. Over 70% of nitrogen was measured in the grain, while only 30% was stored in the residue. Similarly, there existed huge differences in the chemical constitutions between the leaf’s mid-rib and the leaf’s edge. Due to its physiological function in transporting water, the leaf’s mid-rib usually has a higher water content than the other leaf parts [17]. This is also reflected by the phenomenon in which the leaf’s tips and edges turn brown first when the plant is drought-stressed.

The nutrient and stress patterns of the leaf have been proven to contain unique features for plant phenotyping studies. One of the most common applications of leaf distribution features is the determination of the SPAD reading position on the leaf. Due to the unique chlorophyll pattern, researchers need to find the best representative sampling point for the SPAD measurement. It was suggested that a position one third of the distance from the rice leaf’s base to the leaf’s tip was the best position to represent the chlorophyll content of the entire lead blade with low variance of measurements [14]. Moreover, different nutrient deficiencies show obvious visible distribution patterns, even from RGB images. A report from DuPont Pioneer (IA, USA) showed that, in the RGB images of corn leaves, specific patterns could be easily observed and used for differentiating nutrient deficiencies such as nitrogen, phosphate, magnesium, and potash [18]. Therefore, it is critically important to develop a finer level of image processing algorithm for nutrient and stress distribution analysis across the leaf, which could provide better measurements.

Some researchers have realized that the distribution of spectra on the leaf contains useful information for improving the phenotyping quality [13,14,19]. The simplest proposed idea was to measure multiple significant points along the leaf’s blade for a more comprehensive evaluation of the plant. Vig [20] suggested measuring five points while using a SPAD meter: the leaf apex, the first quarter of the leaf blade, the center of the leaf blade, and the left and the right of the leaf base. This multipoint measurement method was proven to suffer less variance and be more accurate in describing the whole plant. Similarly, Hu [13] explored the changes in SPAD readings along both leaf directions: (1) from the leaf base to the leaf tip; and (2) from the main vein to the leaf margin. For both directions, the SPAD readings for the nitrogen-stressed plants were observed to have larger variations compared to the healthy plants, which could be used for diagnosing nutrient deficiency. In current leaf distribution studies, the whole leaf is simply divided into different parts. By comparing the averaged measurements between different leaf parts, it is easy to find high variability from the leaf collar to the leaf tip [20,21]. However, these distribution studies are still at early stages. Researchers are trying to develop simple empirical solutions, which can not efficiently utilize all the distribution features along the leaf. The more detailed distribution information across the leaf is still missing, which may contain valuable signals for phenotyping analyses.

Instead of the traditional empirical methods, a supervised machine learning (ML) approach would be a more promising alternative to analyze and interpret the distribution of spectra on the leaf [22]. Due to the mechanisms of ML models, they can learn from data and, based on the learning, they can make the best predictions [23]. In fact, significant work has been done in similar plant phenotyping analyses, such as predicting nutrient contents [24,25], estimating the plant field [26], accessing biotic or abiotic stresses [27], and classifying desired traits [28,29]. However, analyses of the distribution features of leaves using supervised ML models has rarely been conducted. In this paper, the main objective is to introduce a new algorithm to analyze the nutrient and stress distributions on the leaf’s surface, at the pixel level, with machine learning approaches. We took the normalized difference vegetation index (NDVI), one of the most popular vegetative indices [30,31], as an example: Hyperspectral images of top-collared corn leaves were collected. The images were processed to generate high resolution NDVI heatmaps of each individual leaf. NDVI distribution patterns along the leaf’s mid-rib direction were successfully modeled with selected machine learning algorithms. The NDVI distribution pattern was then combined with the spectral information for a more precise assessment of the nitrogen stress level. The result showed that, compared with the traditional algorithms which use an averaged spectrum, the new distribution analysis algorithm was able to improve the phenotyping quality significantly.

## 2. Materials and Methods

### 2.1. Experimental Design

A field experiment was designed and carried out with 64 corn plants (B73xMo17) and 32 corn plants (P1105AM) in the Agronomy Center for Research and Education field #9D of Purdue University (40°28’14.3”N, 86°59’40.2”W, 4540 US-52, West Lafayette, IN 47906) on 17 July 2018. Plants were grown in four plots. Each plot was about 5.5 m in length and 1.5 m in width. The plots of the B73xMo17 genotype were equally divided into two groups: a high nitrogen treatment group (H), treated with 250 kg/ha; and a low nitrogen treatment group (L), treated with 0 kg/ha. The P1105AM genotype plants were all high nitrogen treated with 250 kg/ha There were four plots of replicates for each group, and eight plants were evenly selected from each plot as samples. In total, each group had 32 replicates. In this study, the B73xMo17 plants were labeled as the control group used for the model construction. The P1105AM plants had a high nutrient use efficiency property [32], and they were labeled as the validation group for the performance evaluation.

### 2.2. Handheld Hyperspectral Device and Plant Sampling

**Figure 1:**
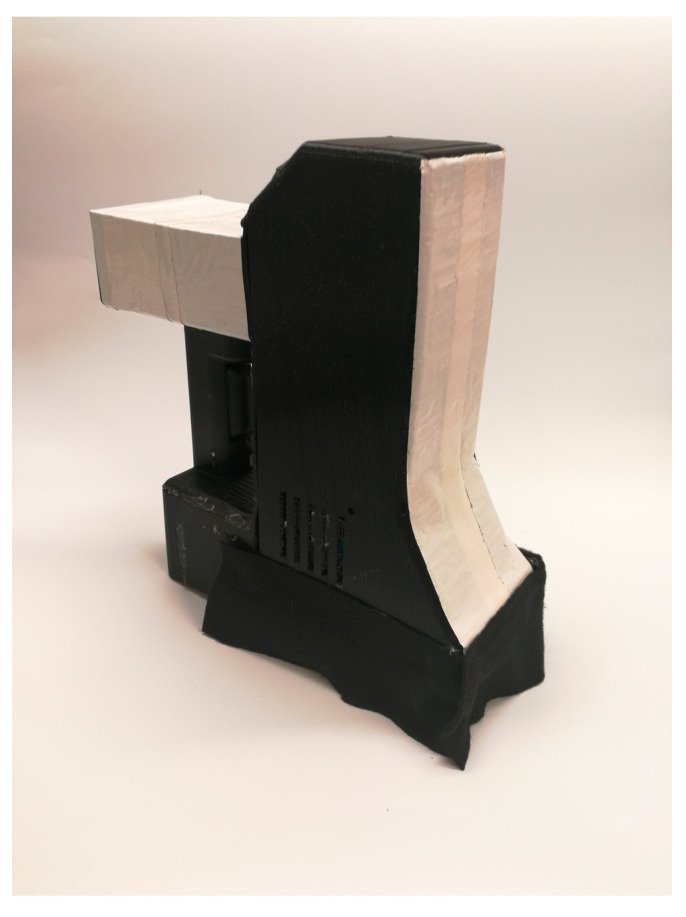
Handheld hyperspectral imaging scanner (LeafSpec). The hardware of LeafSpec consists of four main components: a camera sensor, a scanning mechanism, a light box, and an ARM® based microcontroller.

The leaf-scale hyperspectral images of the corn plants in this experiment were collected with a portable hyperspectral corn leaf imager, LeafSpec, developed by the Purdue Phenotyping Lab group [33]. As shown in Figure 1, the LeafSpec device takes a hyperspectral image of the entire leaf by smoothly sliding the leaf through it. The imaging process takes about 5 s for each specimen [1]. LeafSpec has a spectral range of 450 to 900 nm, and its sampling spectral resolution is 0.74 nm. It is a push broom imager with a spatial resolution of 878 pixels on each line (Table 1). The device collects both spectra and morphological data for each leaf. The on-board microcontroller processes the images from the camera and sends the resulting data to a smartphone app in real time. The smartphone app then automatically uploads the geo-referenced phenotyping results (GPS location and timestamp information) to Purdue’s GeoHub GIS system immediately after each measurement for map-viewing purposes. In this experiment, LeafSpec was used to scan the top matured leaf of each sample plant at the V8 stage, when the plant had eight leaves with visible leaf collars, from the leaf collar to the leaf tip.

### 2.3. Image Processing and Segmentation

**Figure 2:**
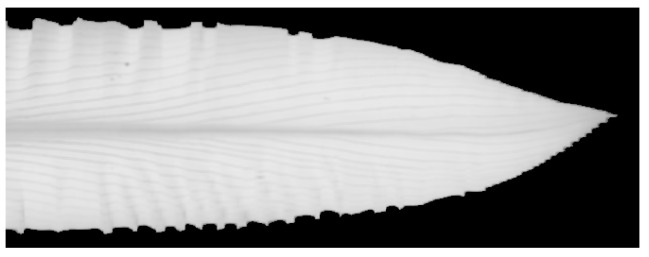
The segmentation result from the raw hyperspectral image. The grey region (plant tissue) was successfully separated from the black region (background).

After data collection, the raw hyperspectral images were first calibrated with a flat strip made with white Teflon material as the reference. The angle of the white reference was perpendicular to the view of the camera and lighting source. The image calibration was performed with the following equation Equation (1):

(1) $$R_{cali}=\frac{R_{raw}-R_{dark}}{R_{white}+R_{dark}}$$

where $R_{cali}$ is the calibrated image, $R_{raw}$ is the raw hyperspectral leaf image, $R_{dark}$ is the dark reference image, and $R_{white}$ is the hyperspectral image of the white reference. Then, the leaf tissue was segmented from the calibrated images using a segmentation procedure with a convolution methodology [12]. A vector of sequential integers from −20 to 20 was multiplied by the reflectance intensity vector from the red-edged region (680–720 nm). By choosing threshold 7 as the boundary between the plant tissue and the background, the plant tissue was successfully segmented, as shown in Figure 2. The spectral features of the leaf tissue (grey region in Figure 2) were used in this study. Due to the high resolution of the leaf images, the detailed spectra distribution features of the leaf were obtained.

### 2.4. NDVI Calculation

Once the hyperspectral leaf images were processed and segmented for all the 64 (B73xMo17) and 32 (P1105AM) corn leaf samples, the Normalized Difference Vegetative Index (NDVI) Equation (2) was calculated for each pixel. This index was reported to be related to the nitrogen status and chlorophyll content of the plant [34,35]. Since NDVI values range from 0 to 1, the jet color bar was set from 0 (blue) to 1 (red) for the NDVI heatmap image.

(2) $$NDVI=\frac{R_{800nm}-R_{650nm}}{R_{800nm}+R_{650nm}}$$

where R_800nm_ and R_650nm_ are the reflectance values of wavelengths 800 nm and 650 nm, respectively [36,37].

One traditional way of evaluating the plant’s nitrogen level is through calculating the average NDVI of each corn leaf Equation (3). The average NDVI was compared with the new stress distribution analysis algorithm’s result.

(3) $$Averaged\;NDVI=\frac{1}{n}\overset{n}{\underset{1}{\sum }}NDVI_{pixel}$$

where n is the total number of pixels from the segmented leaf image, and NDVI_pixel_ is the pixel-level value.

### 2.5. Distribution Feature Estimation and Quantification

#### 2.5.1. Preprocessing of NDVI Images

**Figure 3:**
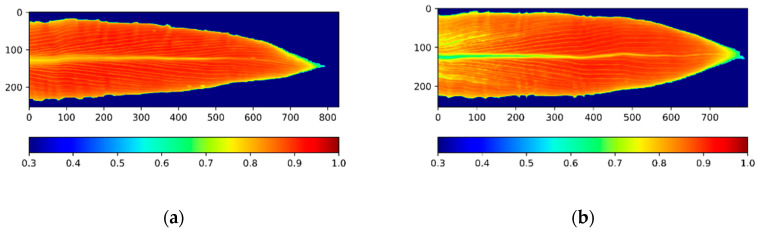
The Normalized Difference Vegetative Index (NDVI) image of the top-collared leaves of corn plants under different nitrogen deficiencies at the V8 stage. (**a**) High nitrogen treatment; (**b**) Low nitrogen treatment. The NDVI image followed the expected impacts from nitrogen stresses.

**Figure 4:**
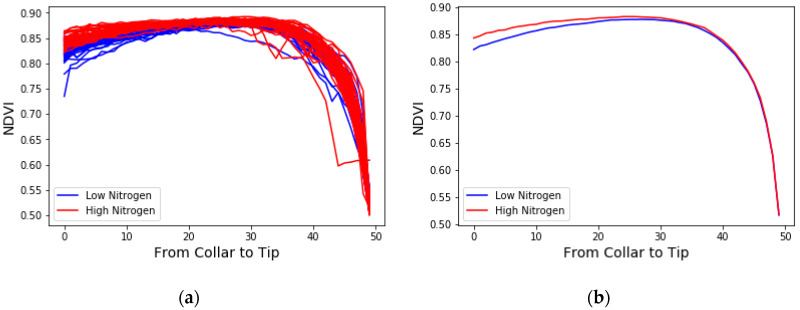
The average NDVI value of each scanning line along the leaf under two nitrogen treatments at the V8 stage. The X coordinate is the rescaled leaf from the leaf collar (0) to the leaf tip (50). (**a**) All 64 corn leaf samples for genotype B73xMo17; (**b**) The average of each nitrogen-stressed group. Red indicates the high nitrogen group, and blue indicates the low nitrogen group.

To efficiently utilize the stress distribution information from the obtained NDVI images, we mainly focused on the mid-rib direction of the leaf (the horizontal direction (X) in Figure 3), which had the major variance in the stress distribution [20,38]. After averaging the NDVI values along each scanning line (the vertical direction (Y) in Figure 3), a further rescaling process was applied in order to rescale all the leaf images to the same length. Since the original leaf samples had different lengths, it was important to rescale them before comparing their distribution patterns. After rescaling, we equally divided the leaf image into 50 sections along the mid-rib direction (X) and calculated the average NDVI for each section. The changes in the NDVI across the 50 sections are shown in Figure 4. Figure 4 shows the different distribution patterns from the different stressed groups: the NDVI value gradually increased along the mid-rib direction and suddenly dropped near the leaf tip. Differences in the NDVI’s changing ratio at different sections were observed between the different stressed groups.

#### 2.5.2. Application of Machine Learning Algorithms

**Figure 5:**
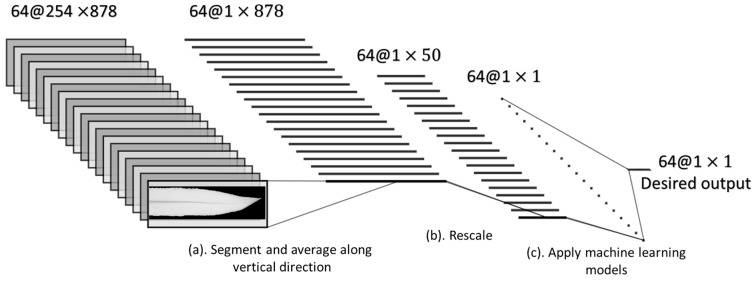
Overall schematic of the distribution feature analysis, from the raw NDVI image to the final prediction. It contains three major steps: (**a**) Segment the leaf and average along the vertical direction; (**b**) Rescale; (**c**) Model with the machine learning (ML) methods. Here, 64 @ 254 × 878 means that the original size of the NDVI image is 254 × 878, with 64 replicates for the model training.

After preprocessing, the NDVI distribution pattern across the X direction was utilized for the improved assessment of the nitrogen stress level by training machine learning algorithms. The overall schematic of the distribution feature analysis procedure is shown in Figure 5.

The NDVI images were rescaled to 1×50 vectors containing the distribution information along the leaf’s midrib direction. With 64 training samples from genotype B73xMo17, the final input data constituted a 64×50 matrix (Figure 5b). The desired output matrix was the nitrogen treatment (stressed vs. no stress). In this study, we had two nitrogen treatments: the high nitrogen treatment group (H), treated with 250 kg/ha, and the low nitrogen treatment group (L), treated with 0 kg/ha. The nitrogen treatments were labeled 1 and 0, representing high and low nitrogen treatment, respectively.

Finally, we obtained a 64×50 input matrix and a 64×1 output vector (Figure 5c), and we selected the four most commonly used supervised machine learning algorithms from the Scikit-learn library [39] and denoted them as the regression models (Table 2). The prediction results of the regression models were then used to evaluate the nitrogen stress level. In order to avoid overfitting issues, we conducted a 10-fold cross-validation to evaluate each model’s performance. The data were randomly divided into 10 subsets. The nine subsets were used to train the regression models and the left subset was used as the validation set. The whole validation process was repeated 10 times. The final estimation error was averaged over all 10 trails for each model.

#### 2.5.3. Hyperparameter Optimization

Hyperparameter optimization is the process that is used to maximize a supervised ML model’s performance without overfitting, and it is vital in order to enhance the model’s performance [23]. For optimizing the selected four ML models (AdaBoost, Logistic Regression, Partial Least Squares Regression (PLSR), and Random Forest), the grid search was selected and performed for the best tuning parameter values. Starting from the default setting in the Scikit-learn library, a set of candidates for the tuning parameter values are specified and then evaluated for each of the methods. In consideration of the efficiency and accuracy of the model searching, we only chose one hyperparameter of each ML method for the grid search. Taking Random Forest as an example, the maximum depth was chosen as the tuning parameter from values in a range of 1 to 10. During the whole parameter tuning process, each of the trained models was 10-fold cross-validated to prevent overfitting. Finally, the best hyperparameter for each model was determined based on the lowest cross-validated root mean square error (RMSE). RMSE is interpreted as the absolute measure of fit, which is commonly used in optimizing machine learning models. It is a good measure of how accurately the model predicts the response, and it is the most important criterion for fit if the main purpose of the model is prediction [44].

#### 2.5.4. Metrices for Model Evaluation

To evaluate the algorithms’ performance, we conducted the two-sample t-test on the predictions between the low and high nitrogen groups. We hypothesized that by incorporating the NDVI’s spatial distribution information, the new models’ results could more clearly separate the nitrogen treatments, compared with the traditional averaged NDVI.

In addition to the nitrogen stress assessment, the new model’s prediction results were also used to separate the images from two different corn genotypes: (1) B73xMo17 (control); (2) P1105AM (high nutrient use efficiency). P1105AM is a commercial corn product from Pioneer, and it has been proven to have a high nutrient use efficiency in many studies because it can contribute to deep root system development and increased nutrient absorption by the plant [32]. Similarly, the two-samplet-test result was used to compare the new model’s predictions with the conventional averaged NDVI value.

### 2.6. Software and Computation

In this study, the supervised machine learning analysis and figure plotting were performed in the Python version 3.7.2 software environment [45]. Machine learning algorithms were carried out with the package from the Scikit-learn machine learning library [39]. The dataset was analyzed and manipulated using Pandas [46] and Numpy [47]. The figures were drawn with Matplotlib [48]. All the computations were run on an HP 17 G3 Mobile Workstation (Hewlett-Packard, Palo Alto, CA, USA) equipped with 64-gigabytes (GB) of random-access memory (RAM) and a 2.70 GHz Intel® Core™ i7-6820HQ processor and a GPU of Nvidia Quadro P3000.

## 3. Results

### 3.1. The Averaged NDVI Comparison

**Figure 6:**
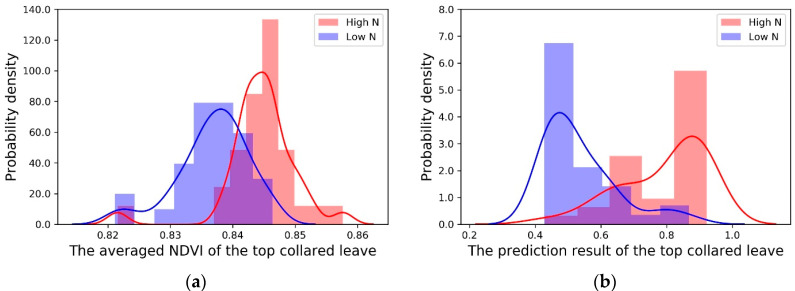
The probability density distribution (PDE) of top-collared leaves for different nitrogen treatments: High N (red); Low N (blue). (**a**) The PDE of the averaged NDVI. (**b**) The PDE of the prediction results from the Random Forest algorithm.

The traditional averaged NDVI values from the leaf images were compared between the different nitrogen treatments and genotypes. Based on the two-samplet-test, the high nitrogen group showed a significantly higher NDVI than the low nitrogen group (Table 3). However, the distributions of the averaged NDVIs were largely overlapped. Figure 6a shows the estimated probability density distributions of the averaged NDVI for the two nitrogen treatments by using the kernel density estimate (KDE) [29].

**Figure 7:**
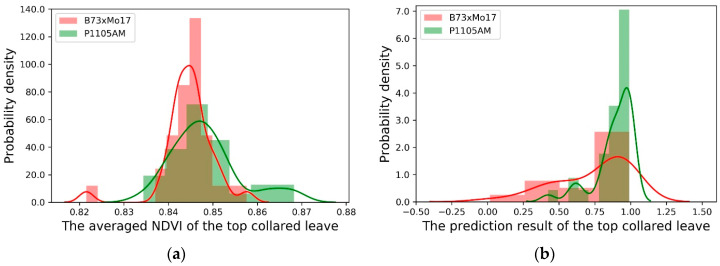
The probability density distribution (PDE) of top-collared leaves for different genotypes: B73xMo17 (red); P1105AM (green). (**a**) The PDE of the averaged NDVI. (**b**) The PDE of the prediction results from the Random Forest algorithm.

Similarly, the P1105AM genotype showed a significantly higher averaged NDVI than the control genotype (Table 4). However, the distribution of the averaged NDVI values between the two genotypes was severely overlapped too (Figure 7a). This would lead to severe type 1 and type 2 errors in classifying the plants, either for nutrient or breeding studies. Therefore, this confirmed that the conventional averaged NDVI method is not satisfactory in this kind of plant analysis study. A finer level of image processing algorithm for nutrient and stress analysis is preferred for improved quality of measurement.

### 3.2. Results of Machine Learning Algorithms

The results of the four supervised learning models are summarized in Table 3. They showed that AdaBoost, Logistic Regression, PLSR, and Random Forest all had smaller two-sample t-test P-values than the averaged NDVI. Among them, Random Forest had the lowest P-value for classifying corn leaves from different nitrogen treatments. Besides this, the standard deviation of the prediction results was used to check the consistency of the model. Random Forest had the lowest and most consistent standard deviation values for both the high and low nitrogen groups. Taking both findings into consideration, the Random Forest algorithm delivered the best performance with the most significant difference and the greatest consistency.

To illustrate the performance of the predictions from the new Random Forest model, the distributions of the prediction results from the two nitrogen treatments are shown in Figure 6b. Compared with the distributions of the traditional averaged NDVI (Figure 6a), the new model’s predictions were able to more clearly separate the two treatments, as shown in Figure 6b. The -log (*P*-value) of the two-sample t-test for the new model’s predictions also increased to 9.519 from 5.845 when the traditional averaged NDVI was used.

### 3.3. Model Evaluation for Different Corn Genotypes

Similarly, the prediction results of the new Random Forest model were also used to differentiate between the two corn genotypes: (1) B73xMo17 (control); (2) P1105AM (high nutrient use efficiency). The statistical results are summarized in Table 4. The results showed that the genotype difference was more significant with the new model’s prediction result (*p*-value = 0.004), while it was less significant with the conventional averaged NDVI (*p* = 0.035). Since the genotype difference is usually less significant in plant breeding studies [49], it indicated that the new model was better at detecting weaker signals compared to the traditional averaged NDVI.

## 4. Discussion and Conclusions

In most of the existing hyperspectral image process algorithms, the spectra across all the plant pixels are averaged in order to predict the plant’s physiological features. Due to the high variance in the nutrient and stress levels between different locations on the same leaf, the averaged spectrum might not provide a satisfactory degree of plant measurement quality.

In this paper, a new hyperspectral image processing algorithm based on Random Forest and leaf-region rescaling was introduced in order to analyze the nutrient and stress distribution patterns along the top-collared corn leaf. NDVI was selected as an example feature, since it is widely used to predict the nutrient status (water, nitrogen content, etc.) of plants, mainly from leaf mesurements [33,34,38]. A handheld hyperspectral scanner called LeafSpec was used to scan and collect the hyperspectral images of the top-collared leaves of 96 corn plants with different nitrogen treatments and genotypes. The NDVI distribution patterns within a single leaf were modeled to predict the plant’s nutrient status. Four machine learning regression models were selected and compared for this purpose. All four models were able to more clearly separate the nitrogen treatments than the traditional averaged NDVI across all the leaf pixels. Among the four models, the Random Forest method showed the best performance. Similarly, the new model for NDVI distribution analysis also outperformed the traditional averaged NDVI in differentiating the leaves from different genotypes as well. The results indicate that although NDVI is a good indicator of the nutrient status of plants, the evalution of nutrient status can be further improved with our distribution analysis algorithum.

Compared with the conventional method, which only considers the averaged value, the analysis of distribution features utilized all the variance information in the captured image. Taking both intensity value and distribution information into consideration, the proposed Random Forest algorithm successfully improves phenotyping quality, with a better sensitivity in detecting the plant’s nutrient conditions. Given the successful results, the new distribution analysis algorithm could potentially be applied to other plant feature measurements as well.

In the future, we would like to collect more diverse leaf samples in order to validate the current trained model, as well as to make the prospective model more robust. Moreover, an exhaustive grid search will be done with more hyperparameters involved. Thus, the potential of the current Random Forest model and other ML models could be fully explored. Meanwhile, subspace clustering approaches, such as deep clustering [50] and structured auto-encoder methods [51], will also be explored. Finally, the deep learning-based approach will be tested, as it could learn to automatically extract the features from the raw images, which might enable it to fully make use of the data.

## Figures and Tables

**Table 1:** Parameters for hyperspectral imaging test.

Parameters	LeafSpec
Camera model	BFLY-U3-05S2M-CS
Spectrograph	Customized
Frame rate (FPS)	20
Exposure time (ms)	50
Spectral resolution	676
Spatial resolution (pixels)	878
Spectral range (nm)	450–900
Scan speed (mm/s)	5.08

**Table 2:** Overview of machine learning algorithms used for this study.

Regression Algorithm	Description	Reference
AdaBoost	Adaptive Boosting (AdaBoost) is a generalized boost method which is an ensemble technique that attempts to create a strong classifier from several weak classifiers.	[40]
Logistic Regression	Logistic Regression is a predictive analysis method usually used when the dependent variable is dichotomous (binary).	[41]
PLSR	Partial Least Squares Regression (PLSR) is a method that performs least squares regression on new components after reducing original predictors to a smaller set of uncorrelated components.	[42]
Random Forest	Random Forest is an ensemble technique performing both regression and classification tasks with the use of multiple decision trees with Bootstrap Aggregation.	[43]

**Table 3:** The results of the averaged NDVI values and the prediction results from different machine learning algorithms for B73xMo17.

Regression Algorithms	Nitrogen Treatment	Samples #	Mean of the Prediction Results	Standard Deviation	-Log (*p*-Value)
Averaged NDVI	High N	32	0.845	0.006	5.845
	Low N	32	0.837	0.006	
AdaBoost	High N	32	0.744	0.267	7.995
	Low N	32	0.281	0.284	
Logistic Regression	High N	32	0.506	0.008	7.066
	Low N	32	0.493	0.009	
PLSR	High N	32	0.738	0.225	9.375
	Low N	32	0.242	0.298	
Random Forest	High N	32	0.727	0.263	9.519
	Low N	32	0.246	0.242	

**Note:** Larger -log10 (*p*-value) means the two-sample *t*-test is more significant.

**Table 4:** The comparison results of the conventional averaged NDVI and the prediction results from Random Forest.

Methods	Corn Genotypes	Nitrogen Treatment	Sample #	Mean	Standard Deviation	*p*-Value
Averaged NDVI	B73xMo17	High N	32	0.845	0.006	0.035
	P1105AM	High N	32	0.848	0.008	
Prediction result from Random Forest	B73xMo17	High N	32	0.727	0.263	0.004
	P1105AM	High N	32	0.886	0.137	

**Note:** Smaller *p*-value means the two-sample *t*-test is more significant.
